# Case Report: Chorea as a Rare Manifestation of Secondary Adrenal Insufficiency

**DOI:** 10.7759/cureus.58353

**Published:** 2024-04-15

**Authors:** Shalesh J Rohatgi, Satish P Nirhale, Prajwal M Rao, Pravin U Naphade, Pranit D Khandait

**Affiliations:** 1 Neurology, Dr. D. Y. Patil Medical College, Hospital and Research Centre, Dr. D. Y. Patil Vidyapeeth, Pune, IND

**Keywords:** hyperkinetic disorder, chorea due to adrenal insufficiency, secondary adrenal insufficiency, euvolemic hyponatremia, chorea

## Abstract

The word "chorea" comes from the Latin word "choreus," which means dancing movement. Chorea is defined as a hyperkinetic movement disorder characterized by uncontrolled, unintended, jerky, brief, irregular, random movements involving the limbs or facial muscles.

Here, we discuss the case of a 48-year-old male with hypothyroidism for two years, which is well-controlled with medication. He presented with behavioral disturbances for the past seven months and choreiform movements affecting all four limbs, his tongue, and his face for the past six months. Investigations revealed hyponatremia and low serum osmolality. An MRI of the brain showed the empty sella sign. Further investigations revealed low levels of adrenocorticotropic hormone (ACTH), prolactin, and testosterone. Considering the diagnosis of chorea with euvolemic hyponatremia due to secondary adrenal insufficiency, the patient was started on tetrabenazine, trihexyphenidyl, oral hydrocortisone, and gradual correction of sodium level. The patient's condition improved during the hospital stay, and he continues to do well in routine follow-ups.

## Introduction

Chorea is a hyperkinetic movement disorder caused by an imbalance between the direct and indirect pathways of the basal ganglia circuit, leading to excessive dopaminergic activity. This results in a loss of inhibitory activity in the pallidum, culminating in chorea [1].

The causes of chorea can be either hereditary or acquired. Acquired causes can result from autoimmune or inflammatory conditions like antiphospholipid antibody syndrome, autoimmune encephalitis, Sydenham's chorea, and systemic lupus erythematosus. Other causes encompass stroke, dopaminergic drugs, infective agents, and metabolic imbalances, such as hypo/hypercalcemia, hypo/hyperglycemia, hypo/hypernatremia, and polycythemia vera. Pregnancy-induced chorea (chorea gravidarum) is also observed. Hereditary causes include Huntington's disease, dentatorubral-pallidoluysian atrophy, neuroferritinopathy, spinocerebellar ataxia types 1, 2, 3, and 17, as well as ataxia-telangiectasia and others [1,2].

Here, we discuss a rare case of a 48-year-old male who presented with behavioral disturbances, generalized chorea, and euvolemic hyponatremia due to secondary adrenal insufficiency.

## Case presentation

A 48-year-old male, an engineer by profession, presented with behavioral disturbances in the form of increased irritability and aggressive behavior for the past seven months. Additionally, he experienced increased daytime sleepiness, which had been gradually worsening. This was also accompanied by involuntary movements involving all four limbs. These movements were uncontrolled and manifested as jerky, brief, irregular, and random movements, including facial and tongue muscles, over the last six months. These movements typically occurred during rest and increased with distracting activities, but they were absent during sleep. There was no history of cranial nerve involvement, sensory-motor weakness, or bowel or bladder dysfunction. Furthermore, there was no history of a sore throat, joint pain, photosensitivity, rash, mucocutaneous ulcers, or pigmentation. There was no history of prior fever, weight loss, jaundice, toxin exposure, or drug intake. His family history was unremarkable. He had a known case of hypothyroidism for the past two years and was well-controlled on medication.

On examination, his pulse was 90/min, and his blood pressure was 90/60 mmHg. There was no pallor, icterus, clubbing, or lymphadenopathy. The Mini-Mental State Examination (MMSE) score was 27/30, and the Frontal Assessment Battery (FAB) score was 14.

Choreiform movements (Video 1), characterized by brief, semi-purposive dancing and non-suppressible movements, were observed more in the lower limbs than in the upper limbs, with involvement of the face and tongue.

**Video 1:** Generalized chorea at presentation

Motor impersistence was noted in the tongue, and the milkmaid grip was present. The ophthalmic examination did not reveal a Kayser-Fleischer (KF) ring, and the fundus examination was normal. Cranial nerve and sensory-motor examinations were within normal limits.

Complete blood count and renal and liver function tests were within normal limits. The peripheral smear was negative for acanthocytes. The random blood sugar level was 88 mg/dL (HbA1C: 5.6%).

Thyroid function test showed triiodothyronine (T3) at 1.19 ng/mL, thyroxine (T4) at 0.52 µg/dL, and thyroid-stimulating hormone (TSH) at 0.96 uIU/mL.

**Figure 1:**
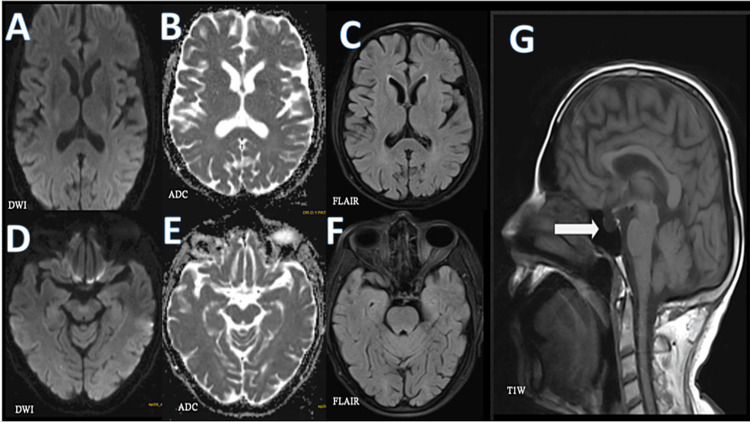
MRI brain revealed an empty sella sign Sections at the level of basal ganglia (A,B,C) and sections at the level of the midbrain (D,E,F) demonstrate corresponding DWI, ADC, and FLAIR images, which show no infracts or lesions. The T1W (G) sagittal image revealed an empty sella sign (marked by a white arrow). ADC, apparent diffusion coefficient; DWI, diffusion-weighted imaging; FLAIR, fluid-attenuated inversion recovery; T1W, T1-weighted

The MRI of the brain (Figure 1) revealed an empty sella sign with no other significant abnormality seen.

Serum electrolyte analysis showed hyponatremia (sodium: 119 mEq/L), with a low serum osmolarity of 256 mOsm/kg (normal range: 275-295 mOsm/kg). Urine osmolarity was 327 mOsm/kg (normal range: 50-1,200 mOsm/kg), and urine sodium was 57 mEq/L (normal range: 20-150 mEq/L).

Serum calcium, magnesium, ferritin, creatine phosphokinase (CPK) total, copper, and 24-hour urinary copper levels were within normal limits. Serum anti-streptolysin-O (ASO) titer was negative.

Cerebrospinal fluid examination showed protein at 32 mg/dL, glucose at 66 mg/dL (corresponding blood sugar level: 100 mg/dL), and two cells, all lymphocytes.

Further workup showed serum cortisol levels of 1.4 nmol/L (normal range: 8-15 nmol/L), adrenocorticotropic hormone (ACTH) at 8.27 pg/mL (normal range: 15-45 pg/mL), serum testosterone at 6.36 ng/dL (normal range: 240-950 ng/dL), and serum prolactin at 0.67 ng/mL (normal range: 3.46-19.4 ng/mL). The serum autoimmune panel was negative.

Thus, this was a case of generalized chorea with euvolemic hyponatremia due to secondary adrenal insufficiency. The patient was started on tetrabenazine 12.5 mg twice daily (BD) and titrated up to 25 mg BD, trihexyphenidyl 2 mg BD, and oral hydrocortisone 10 mg BD. Slow sodium correction was done. Thyroxine supplementation was continued. The patient showed a dramatic improvement in symptoms. The patient is under regular follow-up. After two months, trihexyphenidyl and tetrabenazine were gradually tapered and stopped. The patient is currently on oral hydrocortisone and thyroxine. There are no choreiform movements (Video 2) or behavioral disturbances at present, and there are no new complaints.

**Video 2:** No choreiform movements on follow-up

## Discussion

Chorea is typically present during rest and may increase with distracting activities, but it vanishes during sleep. Generalized chorea involves limbs along with facial muscles, as observed in our patient [1-3]. Behavioral disturbances can be seen in adrenal insufficiency, which was observed in our case as well [4].

An altered sleep cycle has been reported in adrenal insufficiency, which was also present in our case in the form of excessive daytime sleepiness [5]. Hypotension and hyponatremia were seen in our case due to low cortisol levels, which indicate adrenal insufficiency. This was associated with low ACTH levels, suggestive of secondary adrenal insufficiency [6]. On MRI, when the pituitary gland becomes smaller and cannot be detected, this gives rise to the appearance of an “empty sella,” which was observed in our case [7]. The patient was started on oral hydrocortisone, trihexyphenidyl, and tetrabenazine with gradual dose titration [8-10]. To the best of our knowledge, there are no available case reports of generalized chorea with behavioral disturbances as a chronic presentation of secondary adrenal insufficiency.

## Conclusions

Chorea with behavioral disturbances presenting with hypotension and hyponatremia should be evaluated for adrenal insufficiency, as it is one of the treatable causes of chorea. The outcome is promising, as they respond well to hydrocortisone treatment.
